# Validity of estimating physical activity intensity using a triaxial accelerometer in healthy adults and older adults

**DOI:** 10.1136/bmjsem-2019-000592

**Published:** 2019-10-28

**Authors:** Sho Nagayoshi, Yoshitake Oshima, Takafumi Ando, Tomoko Aoyama, Satoshi Nakae, Chiyoko Usui, Shuzo Kumagai, Shigeho Tanaka

**Affiliations:** 1 Omron Healthcare Co Ltd, Muko, Japan; 2 Graduate School of Human-Environment Studies, Kyushu University, Fukuoka, Japan; 3 Faculty of Humanities and Social Sciences, University of Marketing and Distribution Sciences, Kobe, Japan; 4 Department of Nutrition and Metabolism, National Institute of Health and Nutrition, National Institutes of Biomedical Innovation, Health and Nutrition, Shinjuku, Japan; 5 Department of Nutritional Epidemiology and Shokuiku, National Institute of Health and Nutrition, National Institutes of Biomedical Innovation, Health and Nutrition, Shinjuku, Japan; 6 Graduate School of Engineering Science, Osaka University, Toyonaka, Japan; 7 Faculty of Sport Sciences, Waseda University, Tokorozawa, Japan; 8 Center for Health Science and Counseling, Kyushu University, Fukuoka, Japan

**Keywords:** validation, accelerometer, elderly people, physical activity

## Abstract

**Background:**

A triaxial accelerometer with an algorithm that could discriminate locomotive and non-locomotive activities in adults has been developed. However, in the elderly, this accelerometer has not yet been validated. The aim were to examine the validity of this accelerometer in the healthy elderly, and to compare the results with those derived in a healthy younger sample.

**Methods:**

Twenty-nine healthy elderly subjects aged 60–80 years (Elderly), and 42 adults aged 20–59 years (Younger) participated. All subjects performed 11 activities, including locomotive and non-locomotive activities with a Douglas bag while wearing the accelerometer (Active style Pro HJA-750C). Physical activity intensities were expressed as metabolic equivalents (METs). The relationship between the METs measured using the Douglas bag and METs predicted using the accelerometer was evaluated.

**Results:**

A significant correlation between actual and predicted METs was observed in both Elderly (r=0.85, p<0.001) and Younger (r=0.88, p<0.001). Predicted METs significantly underestimated compared with actual METs in both groups (p<0.001). The mean of the errors was −0.6±0.6 METs in Elderly and −0.1±0.5 METs in Younger. The degree of underestimation increased with increasing METs in Elderly (p<0.001). A stepwise multiple regression analysis revealed that predicted METs, age, and weight were related to actual METs in both groups.

**Conclusion:**

The degree of correlation between predicted and actual METs was comparable in elderly and younger participants, but the prediction errors were greater in elderly participants, particular at higher-intensity activities, which suggests that different predicting equations may be needed for the elderly.

**What are the new findings**

- The validity of a triaxial accelerometer developed based on data from aged 20 to 59 years was examined in the healthy elderly.
- A strong correlation was found between actual metabolic equivalents (METs) measured using Douglas bag and predicted METs using a triaxial accelerometer in the healthy elderly.
- In the healthy elderly, predicted METs were underestimated and the degree of underestimation increased with increasing METs.

## Background

The elderly population has increased worldwide.^1^ In particular, in Japan in 2017, over 35.2 million people were aged 65 years or over, constituting 27.7% of the total population and marking a record high.^2^ WHO Global Burden of Disease estimates show that the prevalence of disability, which included impairments, activity limitations, and participation restrictions, increases with age and suggests that more than 46% of people aged 60 years and over have disabilities.^3^ It is well known that moderate to vigorous intensity physical activity (MVPA) plays an important role in preventing disability in elderly people.^4^ The WHO recommendation for physical activity for the elderly demonstrated that people aged 65 years and over should do at least 150 min of moderate-intensity aerobic physical activity throughout the week, or do at least 75 min of vigorous-intensity aerobic physical activity throughout the week, or an equivalent combination of moderate-intensity and vigorous-intensity activity.^4^ Besides, as the most recent study indicated, the elderly have fewer opportunities to reach MVPA,^5^ assessment of light activities, such as household activity other than walking, would also be important. Furthermore, it has been reported that sedentary behaviour of 1.5 metabolic equivalents (METs) or less is related to all-cause mortality in older adults.^6^ From these reports, it seems that measuring the intensity of physical activities is important for physical activity management for elderly people.

Although multiple subjective and objective methods have been proposed for measuring physical activity, questionnaires are a practical, easy to administer to large groups, and cost-efficient method. However, they are prone to either overestimation or underestimation due to inaccurate recall, social desirability, and misinterpretation.^7^ In the elderly, self-reports and questionnaire surveys were found to be affected by the state of health and cognitive function;^8^ therefore, objective methods are considered more desirable for measuring physical activity.

Recently, accelerometers that can measure both total amount of physical activity and intensity level per unit time have been widely used for research as a means of objective assessment of physical activity in free-living conditions. An accelerometer is a device for assessing physical activity using equations developed from the relationship between the acceleration data and the energy consumption obtained by expired gas analysis. Acceleration sensors have been previously mounted mainly with a single vertical axial sensor, but they can be mounted with a triaxial sensor. Moreover, some of the latest studies have been designed to identify the types of physical activity by assessing information derived from the acceleration data.^9–12^

Several types of accelerometers are now marketed, and many devices have been validated in the literature.^13–19^ However, few studies have examined the validity of accelerometer data in elderly subjects, and most have only examined the intensity during walking and MVPA cut-points.^15 16 19^

A triaxial accelerometer with an algorithm that could discriminate locomotive and non-locomotive activities in adults has been developed.^12 20^ However, in the elderly, this accelerometer has not yet been validated. Therefore, the aim were to examine the validity of this accelerometer in the healthy elderly, and to compare the results with those derived in a healthy younger sample.

## Subjects and methods

### Subjects

Twenty-nine healthy Japanese elderly subjects aged 60–80 years (Elderly: 15 males and 14 females) participated in this study. In the initial recruitment, 30 elderly subjects participated, but one was excluded due to lack of data. To clarify the validity of the results in the elderly, 42 Japanese adults aged 20–59 years (Younger: 22 males and 20 females) also participated. A total of 71 subjects participated in this study. The number of subjects was determined based on a previous study.^20^ Subjects were recruited from subject recruitment companies and related organisations of researchers. Subjects were recruited with the same ratio of male to female and adjustment for body mass index (BMI) in each age bracket to the standard Japanese BMI. This study was conducted in accordance with the guidelines set by the Declaration of Helsinki.Subjects were excluded from the study if they had any contraindication for exercise or if they were physically unable to complete the activities.

### Anthropometric measurements

Body weight was measured using a digital scale to the nearest 0.1 kg, with the subjects dressed in light clothing. Barefoot standing height was measured to the nearest 0.1 cm using a wall-mounted stadiometer. BMI was calculated as body weight (kg) divided by height squared (m^2^).

### Experimental protocol

Subjects visited the laboratory after fasting for 12 hours or more in the morning of the day of the experiment. After anthropometric measurements, they performed 11 sequences of normal daily activities with a face mask and Douglas bag while wearing a triaxial accelerometer on the right side of the waist in a controlled laboratory setting. The activities were selected based on a previous study^20^ and several normal daily activities for the elderly were added. The selected activities were as follows: resting in the sitting position as a resting metabolic rate (RMR), filing of documents in a sitting position, filing of documents in a standing position, wiping down, dish washing, hanging and taking in the laundry, vacuuming, radio calisthenics, slow walking (Elderly: 50 m/min, Younger: 55 m/min), normal walking (both groups: 70 m/min) and fast walking (Elderly: 90 m/min, Younger: 100 m/min) on a track. These activities were chosen as representative activities of daily life. The subjects were permitted to consume only water during the experiment. They were instructed to lie down quietly for 30 min, and then RMR in the sitting position for 7 min twice or more. The other activities were subsequently performed for 4–8 min. There was sufficient rest between the activities to eliminate any carry-over effects from one activity to the next. The expired air during each activity from each subject was collected under a steady state in the last 1.5–7 min.

### Indirect calorimetry

During each activity, the expired air was collected in a Douglas bag. Expired O_2_ and CO_2_ gas concentrations were measured using a mass spectrometer (ARCO-2000; Arco System, Kashiwa, Japan), and gas volume was calculated using a certified dry gas metre (DC-5; Shinagawa, Tokyo, Japan). For each measurement, the gas analyser was initially calibrated using a certified gas mixture and atmospheric air. The energy expenditure (EE) was estimated from VO_2_ and VCO_2_ using Weir’s equation.^21^ Reference MET values were calculated as the EE during the activities divided by the measured RMR.

### Triaxial accelerometer

An Active style Pro HJA-750C (ASP) (Omron Healthcare Co., Ltd. Kyoto, Japan) was used in this study. The HJA-750C is a successor to the HJA-350IT, the principles and the validity were reported in previous studies.^12 20^ Both models were mounted with the same acceleration sensor (LIS3LV02DQ; ST-Microelectronics, Geneva, Switzerland) and algorithm^12 20^ (see figure 1 in ref. 20 for the workflow of the algorithm). The device, a Micro Electro Mechanical Systems-based triaxial accelerometer, measured 52×40×12 mm, weighed approximately 23 g, including the battery. Triaxial acceleration was measured with a sensitivity of 3 mG at a sampling rate of 32 Hz. Each of the three signals from the triaxial accelerometer was passed through a high-pass filter with a cut-off frequency of 0.7 Hz to remove the gravitational acceleration component from the signal. The integral of the absolute value of each three axes acceleration signals was calculated over 10 s intervals. This device uses three equations to calculate the intensity of activity according to the type of activity. The equations and their validity were previously described.^12 20^

### Statistical analysis

All values are presented as means and SD. Differences were considered to be significant if the p-value was less than 0.05. Elderly and Younger were compared using an unpaired t-test. The relationship between the METs measured using the Douglas bag method (DB_METs) and METs predicted using the ASP (ASP_METs) was evaluated using Pearson’s correlation coefficient (r). DB_METs and ASP_METs within a group were compared using the paired t-test. The validity of ASP was expressed as error (ASP_METs–DB_METs), error rate ((ASP_METs–DB_METs)/DB_METs×100)) and error plots. Stepwise multiple regression analysis was also carried out to evaluate factors related to DB_METs. DB_METs was applied as dependent variables, and age, sex, weight, BMI and ASP_METs were applied as independent variables.

All statistical analyses were performed using IBM SPSS Statistics V.24.0 for Windows.

## Results

Physical characteristics and RMR in the sitting position are shown in table 1. Although no significant difference was observed in body weight between the groups, RMR in the sitting position of Elderly was significantly lower than that of Younger (p<0.01).

**Table 1:** Physical characteristics and RMR in the sitting position in Elderly and Younger

	Elderly (60–80 years) (n=29)	Younger (20–59 years) (n=42)
Sex (male/female)	15/14	22/20
Age (years）	72.3±5.5 (61–80)	38.4±10.9 (21–55)***
Height (cm）	158.8±10.5 (139.5–179.0)	165.0±8.8 (148.3–182.1)**
Weight (kg）	60.0±11.2 (43.2–92.9)	61.9±13.8 (32.1–91.8)
BMI (kg/m^2^）	23.8±3.2 (16.7–30.6)	22.6±3.8 (14.6–32.0)
RMR (kcal/min）	0.82±0.13 (0.55–1.24)	0.94±0.18 (0.58–1.44)**

Mean±SD (minimum–maximum). **p<0.01, ***p<0.001.

BMI, body mass index; RMR, resting metabolic rate.

DB_METs during activities in both groups are shown in table 2. All values were significantly higher in Elderly for all daily activities.

**Table 2:** DB_METs during the activities in Elderly and Younger

	Elderly (60–80 years)	Younger (20–59 years)
Filing of documents in a sitting position	1.6±0.2 (1.3–2.0)	1.5±0.2 (1.2–2.7)*
Filing of documents in a standing position	2.3±0.3 (1.8–2.8)	2.1±0.4 (1.4–3.0)*
Wiping down	3.0±0.6 (2.1–4.6)	2.6±0.5 (1.7–4.3)**
Dish washing	2.3±0.3 (1.8–3.2)	2.1±0.4 (1.4–3.1)*
Hanging and taking in the laundry	2.8±0.4 (2.2–3.6)	2.5±0.5 (1.7–3.8)**
Vacuuming	3.4±0.7 (1.9–5.1)	3.0±0.7 (1.9–5.1)*
Radio callisthenics	3.7±0.6 (2.5–4.9)	3.2±0.5 (2.2–4.4)***
Normal walking	4.1±0.6 (3.1–5.8)	3.7±0.5 (2.8–5.3)**
Slow walking	3.6±0.5 (2.9–5.2)	3.3±0.5 (2.4–5.2)
Fast walking	5.1±0.8 (3.3–6.9)	5.1±0.8 (3.7–6.9)

Mean±SD (minimum–maximum). *p<0.05, **p<0.01, ***p<0.001.

Slow walking and fast walking were excluded from statistical analyses because the walking speed differed between the groups.

Slow walking (Younger: 55 m/min, Elderly: 50 m/min), normal walking (both groups: 70 m/min) and fast walking (Younger: 100 m/min, Elderly: 90 m/min).

DB_METs, metabolic equivalents (METs) measured using the Douglas bag method.

**Figure 1:**
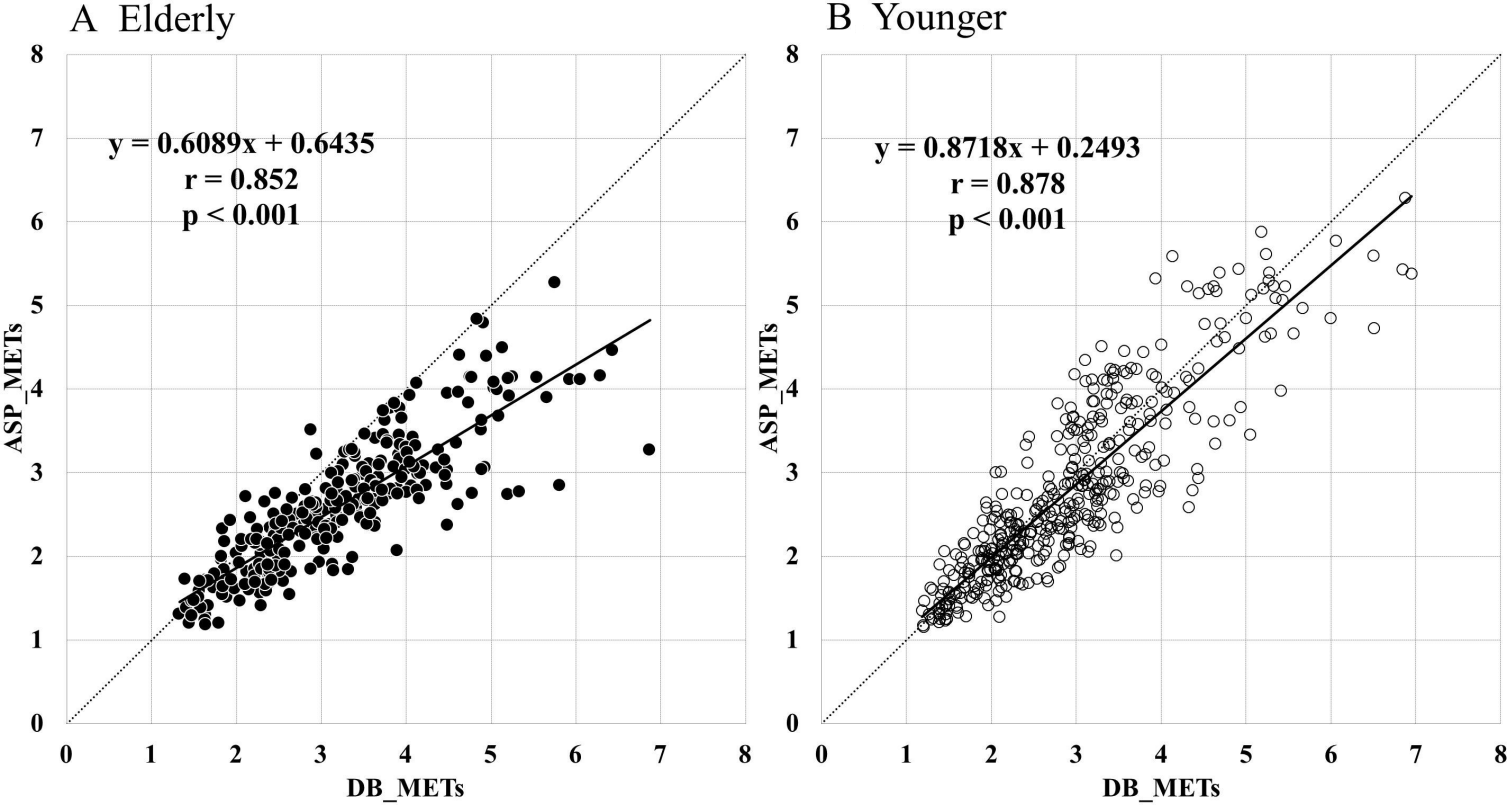
Relationship between the DB_METs and ASP_METs in Elderly (A) and Younger (B). ASP_METs, METs predicted using Active style Pro HJA-750C;DB_METs, metabolic equivalents (METs) measured using the Douglas bag method; Elderly, elderly group aged 60–80 years; Younger, adult group aged 20–59 years.

The relationships between DB_METs and ASP_METs in Elderly (figure 1A) and Younger (figure 1B) are shown separately. A significant correlation was observed in both Elderly (r=0.85, p<0.001) and Younger (r=0.88, p<0.001). ASP_METs significantly underestimated compared with DB_METs in both groups (Elderly: DB_METs=3.2±1.1 METs, ASP_METs=2.6±0.8 METs (p<0.001), Younger: DB_METs=2.9±1.1 METs, ASP_METs=2.8±1.1 METs (p<0.001)). In addition, the mean of the errors was −0.6±0.6 METs in Elderly and −0.1±0.5 METs in Younger, indicating significantly greater underestimation of the ASP_METs for Elderly (p<0.001).

**Figure 2:**
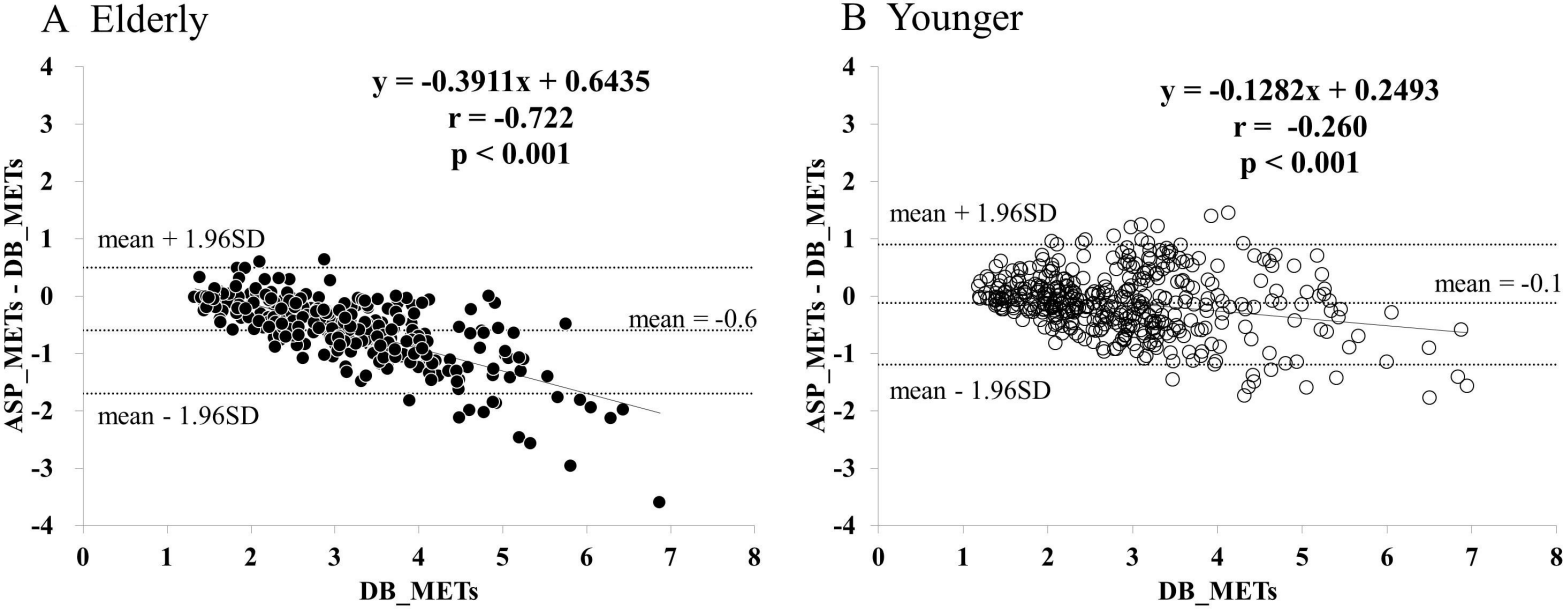
Error plots of ASP_METs in Elderly (A) and Younger (B). ASP_METs, METs predicted using Active style Pro HJA-750C;DB_METs, metabolic equivalents (METs) measured using the Douglas bag method; Elderly, elderly group aged 60–80 years;Younger, adult group aged 20–59 years.

The error plots of ASP_METs (ASP_METS – DB_METs) are shown in figure 2. The degree of underestimation by the ASP increased with increasing METs in Elderly (p<0.001).

The errors and error rate of the ASP_METs compared with the DB_METs in both groups are shown in table 3. The estimated error was significantly higher in Elderly for all activities except for filing documents while sitting or standing.

**Table 3:** Errors (error rates) of ASP_METs during the activities in Elderly and Younger

	Elderly (60–80 years)	Younger (20–59 years)
Filing of documents in a sitting position	−0.1±0.2	0.0±0.2
	(−5.3±11.0%)	(−0.4±13.8%)
Filing of documents in a standing position	0.0±0.3	0.1±0.4
	(−0.8±12.5%)	(6.0%±18.1%)
Wiping down	−0.5±0.4	−0.2±0.5**
	(−14.4±11.9%)	(−4.4±15.4%)
Dish washing	−0.6±0.3	−0.4±0.3**
	(−25.4±9.3%)	(−18.0±11.2%)
Hanging and taking in the laundry	−0.5±0.3	−0.3±0.4**
	(−19.1±9.9%)	(−10.4±13.2%)
Vacuuming	−0.7±0.5	−0.2±0.6**
	(−19.4±12.6%)	(−4.2±18.7%)
Radio callisthenics	−1.0±0.5	−0.5±0.4***
	(−27.7±8.8%)	(−15.9±11.8%)
Normal walking	−0.8±0.7	0.2±0.6***
	(−17.6±13.3%)	(7.7%±15.5%)
Slow walking	−0.7±0.6	0.2±0.5
	(−19.2±12.5%)	(7.5%±16.1%)
Fast walking	−1.0±0.8	−0.1±0.8
	(−18.0±12.2%)	(−0.2±15.2%)

Mean±SD, **p<0.01, ***p<0.001.

Slow walking and fast walking were excluded from statistical analyses because the walking speed differed between the groups.

Slow walking (Younger: 55 m/min, Elderly: 50 m/min), normal walking (both groups: 70 m/min) and fast walking (Younger: 100 m/min, Elderly: 90 m/min).

ASP_METs, METs predicted by Active style Pro HJA-750C.

Stepwise multiple regression analysis was performed to evaluate factors related to DB_METs. Age, sex, weight, BMI and ASP_METs were applied as independent variables. As a result, DB_METs in both groups were found to be related to ASP_METs, age and weight (table 4). Since age was selected prior to body weight by stepwise analysis, the influence of age on the prediction of DB_METs was obvious in Elderly and the partial regression coefficient of age in the model 3 for Elderly was significantly greater than that for Younger (p<0.01).

**Table 4:** Results of the multiple regression analysis in Elderly (A) and Younger (B)

(A) Elderly									
		Partial regression coefficient	SE	Standardised coefficient	T-value	Significance probability	R^2^	Adjusted R^2^	Significance probability
Model 1	(Constant)	0.106	0.117		0.906		0.725	0.724	***
	ASP_METs	1.191	0.043	0.852	27.483	***			
Model 2	(Constant)	−2.017	0.446		−4.523	***	0.747	0.745	***
	ASP_METs	1.198	0.042	0.856	28.719	***			
	Age	0.029	0.006	0.147	4.918	***			
Model 3	(Constant)	−3.058	0.513		−5.962	***	0.759	0.757	***
	ASP_METs	1.201	0.041	0.859	29.486	***			
	Age	0.034	0.006	0.171	5.745	***			
	Weight	0.011	0.003	0.115	3.843	***			

(B) Younger									
		Partial regression coefficient	SE	Standardised coefficient	T-value	Significance probability	R^2^	Adjusted R^2^	Significance probability
Model 1	(Constant)	0.445	0.071		6.298	***	0.77	0.77	***
	ASP_METs	0.884	0.024	0.878	37.268	***			
Model 2	(Constant)	−0.309	0.133		−2.32	^*^	0.792	0.791	***
	ASP_METs	0.885	0.023	0.879	39.187	***			
	Weight	0.012	0.002	0.147	6.563	***			
Model 3	(Constant)	−0.504	0.143		−3.521	***	0.798	0.796	***
	ASP_METs	0.884	0.022	0.878	39.623	***			
	Weight	0.01	0.002	0.126	5.503	***			
	Age	0.008	0.002	0.079	3.44	**			

Dependent variable: METs measured using Douglas bag method (DB_METs).

Independent variables: age, sex, weight, body mass index (BMI) and METs predicted using Active style Pro HJA-750C (ASP_METs).

*p<0.05, **p<0.01, ***p<0.001.

ASP_METs, METs predicted by Active style Pro HJA-750C.

## Discussion

In this study, METs during various activities, including household activities and walking, were evaluated in healthy elderly and younger adults using an ASP. As a result, a strong correlation was observed between ASP_METs and DB_METs in both Elderly and Younger. A significant difference was observed in ASP_METs compared with DB_METs between both groups, and the error of ASP_METs in Elderly was higher than in Younger. ASP_METs significantly underestimated versus DB_METs in Elderly. Among activity types, the errors were approximately 5% for light activities, such as filing of documents while sitting or standing, in both groups, but they increased to approximately 20% in Elderly for more intense activities of 3 METs or higher.

The accelerometer used in this study has an algorithm based on data from men and women aged 20–59 years.^12 20^ It has been reported that the relationship between DB_METs and synthetic acceleration in children aged 6–12 years was different from that in adults,^22^ but the validity of this accelerometer in the elderly aged 60 years and above is unclear. In the study by Park *et al*^19^ using the older HJA-350IT model of the HJA-750C, the validity of this accelerometer during walking was evaluated in elderly subjects aged 65 years and above. As a result, METs predicted by HJA-350IT were underestimated compared with that measured using the Douglas bag method in healthy elderly people and the error in measurements was approximately 14%–17%. In the present study, the METs during walking were similarly underestimated by approximately 17%–19% in Elderly. For activities other than walking, the errors of measurement were similar to those during walking.

There are a few previous studies that examined the cut-off value of MVPA using ActiGraph for the elderly subjects.^15 16 23 24^ Hooker *et al*^24^ investigated the 3 METs cut-points measured using an Actical activity monitor during walking and household activities. They demonstrated that the 3 METs cut-point were lower in the ≥65 years old group than in a younger group (45–65 years), and that the accelerometer underestimated the activity level of elderly people. Corbett *et al*^16^ evaluated the association between ActiGraph GT1M activity monitor counts and METs during walking in older adults (70–90 years). They demonstrated that the accelerometer may misclassify MVPA according to the traditional 2020 counts/min cut-point, and that there is a need to consider lower accelerometer activity count thresholds for assessing MVPA in the older adult population. Thus, previous studies have suggested that METs in elderly people estimated using an accelerometer was lower than the actual measured value during walking. Therefore, in the present study, we evaluated METs during activities other than walking, such as household activities, and obtained similar results. However, Chen *et al*^5^ demonstrated that the elderly, especially people older than 75 years old tended to spend less time in MVPA compared with middle-aged people. Thus, underestimation of ASP_METs in MVPA activities would not have a large impact on evaluating total daily EE in the elderly.

ASP_METs were underestimated in Elderly and DB_METs in Elderly were higher than those in Younger during the same activity. This result was considered to be related to a decrease in RMR in the sitting position and increased energy costs of exercise. In previous studies, it was demonstrated that RMR was lower in elderly people than in young people due to several factors, including ageing and a decrease in lean body mass.^25 26^ In the present study, RMR was also lower in Elderly than in Younger, although these two groups had a similar average body weight. On the other hand, some activities, such as walking, are weight-dependent rather than RMR-dependent.^27^ Moreover, regarding the energy cost of exercise, Mian *et al*^28^ demonstrated that the metabolic cost of average speed walking of elderly people was 31% higher than in younger people due to increasing antagonist muscle coactivation. Probably for these reasons, DB_METs were higher in Elderly, which caused underestimation of ASP_METs.

To evaluate factors related to DB_METs, stepwise multiple regression analysis was performed using DB_METs as the dependent variable. As a result, ASP_METs, age and weight were extracted as factors in both groups. In particular, the influence of age on the prediction of actual METs was significantly greater in Elderly. For example, the partial regression coefficient of age for Elderly indicates that a difference of 10 years old leads to an error of 0.34 MET value. Ortega and Farley^29^ demonstrated that the energy cost during walking was higher in elderly people than in young people, and the difference between the two groups was 14% at minimum speed but 34% at maximum speed, and increasing with the walking speed. In another study, the energy cost during walking was high in obese or overweight elderly people, and excess weight was found to be related to an increase in energy cost in the elderly.^30^ These factors, especially age, may be cause for the different relations between ASP_METs and DB_METs in Elderly and Younger. However, although it is tentative, ASP_METs obtained for Elderly can be adjusted using regression coefficients indicated in table 4.

### Limitations

This study has some limitations. The first is that the subjects were elderly people capable of walking without assistance. Elderly people have widely varying physical abilities, and as the importance of physical activity measurement for the elderly is growing with recent population ageing, studies on elderly people with low physical function levels for who find it difficult to walk without assistance are also necessary. The second was that we could not provide sufficiently robust correction formulae. We could adjust ASP_METs using results obtained by the regression analysis, but the regression coefficients from 29 elderly may not be sufficiently robust. Thus, it is necessary to evaluate in a larger number of subjects. The third was that the maximum age of participants was 80 years. As the number of people older than 80 years is increasing in Japan, validation for such individuals is necessary. The last was the lack of information on body composition in this study. More detailed body composition measurements (eg, Dual energy X-ray absorptiometry) would have benefited to better understand whether the differences in error by age were truly age-related or simply from differences in fat-free mass between elderly and younger participants.

## Conclusions

In this study, METs predicted using a triaxial accelerometer and METs measured using the Douglas bag method during the same activities were compared between elderly and younger participants. A strong correlation was found between predicted METs and actual METs in both groups. A significant difference was observed in predicted METs compared with actual METs in both groups, especially in elderly participants. Thus, it should be kept in mind that predicted METs significantly underestimated against actual METs in the elderly. In addition, the greater underestimation for higher-intensity activities was noted in Elderly. A stepwise multiple regression analysis revealed that predicted METs, age and weight were related to actual METs in both groups.

The degree of correlation between predicted and actual METs was comparable in elderly and younger participants, but the prediction errors were greater in elderly participants, particular at higher-intensity activities, which suggests that different predicting equations may be needed for the elderly.
